# High‐Dose Ascorbic Acid Combined With Dihydroartemisinin Inhibits Lung Adenocarcinoma Malignancy by Inducing Ferroptosis via SLC7A11/GPX4 Pathway

**DOI:** 10.1111/jcmm.70993

**Published:** 2025-12-26

**Authors:** Lan Li, Fei Lu, Sisong Shu, Xiao Jiang, Han Lu, Ke Cao, Zhengting Chen, Jingyan Gao, Mengyuan Liu, Li Chang, Wenhui Li

**Affiliations:** ^1^ Department of Radiation Oncology The Third Affiliated Hospital of Kunming Medical University/Yunnan Cancer Hospital/Peking University Cancer Hospital Yunnan Kunming Yunnan People's Republic of China; ^2^ Key Laboratory of Lung Cancer Research of Yunnan Province Cancer Institute of Yunnan Cancer Hospital Kunming Yunnan People's Republic of China; ^3^ Department of Oncology Tongshan County People's Hospital Xianning Hubei People's Republic of China; ^4^ Department of Oncology The Third Affiliated Hospital of Zhengzhou University Zhengzhou Henan People's Republic of China

**Keywords:** ascorbic acid, dihydroartemisinin, ferroptosis, lung adenocarcinoma, SLC7A11/GPX4

## Abstract

This study evaluated the effect of ascorbic acid (AA) combined with dihydroartemisinin (DHA) in lung adenocarcinoma (LUAD) and the underlying mechanisms to determine whether this combination therapy provides a new therapeutic direction for the treatment of LUAD. The CCK‐8, colony formation and transwell assays were used to assess the vitality, proliferation, invasion and migratory capabilities of LUAD cells after various treatments. Furthermore, a xenograft study was performed to assess the effects on tumour inhibition. Transmission electron microscopy (TEM) was used to investigate the changes in mitochondrial architecture in LUAD cells. Additionally, the levels of reactive oxygen species (ROS), divalent iron, malondialdehyde (MDA), mitochondrial membrane potential, and glutathione (GSH) in LUAD cells were quantified using a detection assay kit. GPX4 and SLC7A11 expression levels were assessed using immunohistochemistry, western blotting, and quantitative polymerase chain reaction. The study results showed the inhibitory effect of AA plus DHA on the viability and progression of tumour cells in vitro; the combined therapy reduced cell proliferation, increased cell death, restricted cell invasion and migration, and significantly reduced tumour development in vivo. Furthermore, we observed an excess of iron inside cells, accumulation of ROS, over‐expression of MDA, a reduction in the mitochondrial membrane potential, and depletion of GSH in response to combined therapy. Three ferroptosis‐related inhibitors partially reversed AA plus DHA‐induced cell death. TEM showed changes associated with ferroptosis in the mitochondria. In addition, the administration of AA with DHA reduced the expression of SLC7A11 and GPX4. Finally, the abovementioned effects of ferroptosis could be regulated by influencing the SLC7A11 gene and GPX4. To our knowledge, this is the first study to show that AA and DHA induced ferroptosis in LUAD via the SLC7A11/GPX4 signalling pathway.

**Abbreviations**
AA: Ascorbic acid
CI: Combinatorial index
FISH: Fluorescence in situ hybridization
GO: Gene Ontology
HE: Haematoxylin and eosin
KEGG: Kyoto Encyclopedia of Genes and Genomes
LUAD: Lung adenocarcinoma
OS: Overall survival
PBS: Phosphate‐buffered saline
PCR: Polymerase chain reaction
ROS: Reactive oxygen species
SEM: Standard error of the mean
TEM: Transmission electron microscopy

## 1 Introduction

Lung adenocarcinoma (LUAD), the most common lung cancer, is responsible for the most deaths due to lung cancer [1, 2]. Pharmacological treatments are an opportunity to provide potential clinical benefit, including chemotherapy, adjuvant chemotherapy, neoadjuvant chemotherapy, targeted therapy, immunotherapy and others. However, the prognosis of LUAD remains poor, and its mortality rate is still among the highest for cancer, with median overall survival (OS) and 5‐year survival rates of only 1 year and 3.5%, respectively [3, 4]. The diagnosis and treatment of LUAD remain a focus of current research. Targeted therapies are now making great progress in the clinical management of patients with LUAD. Compared with chemotherapy, targeted drugs significantly prolong the survival of patients with LUAD with more precise efficacy, less damage to normal tissues, and fewer adverse effects. However, due to acquired resistance, most patients develop drug resistance 8–13 months after targeted drug therapy, which seriously affects its clinical application and efficacy. Drug resistance can occur in chemotherapy, radiotherapy, targeted therapy, and immunotherapy and is a major obstacle in the clinical prognosis. In addition, in patients with advanced LUAD with poor disease control after multiple lines of therapy, the multilinear nature of the combination of drugs increases the adverse effects. As a result, these patients with poorer health conditions have reduced tolerability and limited drug selectivity. Therefore, the selection of safe and effective drugs for combination therapy is a potential new approach to overcoming drug resistance.

Vitamin C, known chemically as ascorbic acid (AA), has been a vital dietary ingredient for years [5]. However, recent studies have begun to uncover its potential as a treatment agent for human malignancies. The antitumor actions of AA are primarily to stimulate oxidation, induce DNA demethylation, regulate immunity, interfere with metabolism, modify iron homeostasis, obstruct glycolysis, and stop translation of the endoplasmic reticulum [6, 7]. AA can promote reactive oxygen species (ROS)‐induced oxidative stress and cell cycle arrest, upregulate *P53*, reduce ATP levels, and impair mitochondrial function. Alone or combined, it inhibits the NRF‐2, HIF‐1α, NF‐κB, and MAPK/ERK signaling pathways and exerts antitumor effects. In addition, it can also promote cancer cell death via the upregulation of *HMOX1* expression through the AMPK/NRF‐2 pathway. Furthermore, oxidative stress brought on by AA may intensify ferroptosis [8, 9]. In vitro, high doses of AA selectively target and kill cancer cells while maintaining good safety tolerance in vivo [10]. Studies have also suggested that ingestion of high‐dose AA is connected to a decreased risk of various malignancies, such as in the oral cavity, stomach, esophageal, pancreatic, cervix, breast, and rectum cancers [11, 12]. However, the antitumor effect of AA alone is controversial and has not been well confirmed in clinical practice [13, 14, 15, 16, 17]. Recent research has shown that AA can improve the therapeutic effect when combined with chemotherapy, immune‐targeted drugs, and other therapeutic approaches [7]. Therefore, combining AA with other medications may be a better direction for improving efficacy.

According to reports, artemisinin and its metabolites, including artesunate, artemether, dihydroartemisinin (DHA), and artemisinin, possess antitumor and antimalarial properties. DHA is the most vital active metabolite extensively implemented in medical practice. Evidence indicates that DHA inhibits tumour metastasis, encourages DNA damage via the increased generation of ROS, triggers programmed cell death, stops the growth of malignant cells and strengthens immunity against cancer [18]. DHA inhibits epithelial–mesenchymal transition (EMT) by decreasing the production of TGF‐β and reducing the phosphorylation of SMAD2, SMAD3 and AKT. In addition, DHA has been shown to have various anticancer effects by inhibiting NF‐κB, including inhibition of proliferation, induction of apoptosis, and inhibition of tumour metastasis. Regarding immunity, DHA enhances T cell activity and promotes the secretion of perforin, granzyme B, and IFN‐γ. Furthermore, DHA can inhibit the differentiation of THP‐1 cells into M2 macrophages, enhance the phagocytosis of dendritic cells and macrophages, and promote the production of IFN‐γ by T cells, significantly enhancing antitumor immunity. Finally, DHA can promote iron addition and accelerate ferroptosis [19]. Regarding combined therapy, DHA might have a synergistic effect in improving the cytotoxicity of AA toward cancer cells; however, to our knowledge, no study has evaluated the antitumor activity of the combination of AA and DHA, especially in LUAD.

Ferroptosis, a newly discovered form of programmed cell death, can occur due to a lipid peroxide buildup that relies on iron [20]. *SLC7A11*, a member of the solute carrier family, is an essential ferroptosis regulator. Suppression of *SLC7A11* significantly inhibited the growth of tumour cells due to increased ferroptosis [21], while activation of *SLC7A11* positively correlated with tumour progression. Another important regulator of ferroptosis downstream of *SLC7A11* is glutathione peroxidase 4 (*GPX4*), which reduces lipid peroxides [22]. It was observed that in vivo tumour development was inhibited by *GPX4* suppression [23]. Therefore, initiating ferroptosis via the downregulation of *SLC7A11/GPX4* may become a potential target for treating LUAD.

Based on this preclinical investigation, we demonstrate that the combination of AA and DHA suppresses LUAD progression, primarily through the induction of ferroptosis. Our results indicate that the treatment promotes key ferroptotic events—including ROS accumulation, Fe^2+^ elevation, MDA increase, and GSH depletion—likely through inhibition of the SLC7A11/GPX4 axis. While ferroptosis appears to be a central mechanism, we acknowledge that other forms of cell death may also contribute to the observed antitumor effects. These findings offer a preclinical foundation for further exploration of AA‐DHA as a potential therapeutic strategy in LUAD.

## 2 Materials and Methods

### 2.1 Cell Culture and Transfection

The LUAD cell lines A549 (cat. no. CL‐0016), H1299 (cat. no. CL‐0165) and LLC (cat. no. CL‐0140) were provided by the Lung Cancer Laboratory of Yunnan Tumour Hospital, which were acquired from Procell (Wuhan Pricella Biotechnology Co. Ltd.). All cell lines were authenticated by their suppliers through STR profiling. A549 and H1299 cells were cultured in RPMI‐1640 medium, and LLC cells were cultured in DMEM. All media were supplemented with 10% fetal bovine serum. Cells were incubated at 37°C in a humidified 5% CO_2_ incubator. GenePharma produced the oe‐*SLC7A11*, oe‐NC, sh‐*SLC7A11*, and sh‐NC. The GigaGene Lentiviral Vector System consists of the GV Lentiviral Vector Series, the pHelper 1.0 vector, and the pHelper 2.0 vector triple plasmid. It's a 3rd‐generation lentivirus. The titers (TU/mL) of SLC7A11‐RNAi [1], SLC7A11‐RNAi [2], sh‐empty, oe‐SLC7A11 and OE‐empty were 2E+9, 1E+9, 1E+9, 1.5E+9 and 5E+8, respectively. The cell line was packaged as HEK293T (Genechem Co. Ltd.), and then the prepared DNA solution was mixed with E‐Trans Transfection Reagent (Cat. No. GRCT105, Genechem), and incubated for 15 min at room temperature, and then slowly added dropwise to the culture medium of HEK293T cells, and then cultured in a cell culture incubator at 37°Cwith 5% CO_2_ after mixing. After 6–8 h, the medium containing the mixture of transfection system was discarded and washed once by adding PBS liquid, slowly adding cell culture medium containing serum, and incubating at 37°C with 5% CO_2_ incubator for 48 h, the supernatant of cells was collected, and then centrifuged at 25000 rpm in an ultracentrifuge for 2 h to extract the viral liquid. HEK293T cells were transfected by adding infectious virus and lentiviral reagent HitransG A (Cat. No. REVG004, Genechem), and cultured at 37°C in a cell culture incubator containing 5% CO_2_, and the medium was replaced with fresh medium after 24 h of infection, and the transfection efficiency was 80% by looking at the fluorescence under the fluorescence microscope at 72 h after infection. Subsequent over‐expression and shRNA transfections were then performed following comprehensive reference instructions. The required viral titre was calculated according to the formula after obtaining the corresponding lentivirus. For cell culture, 5 × 10^4^ cells/well were evenly distributed into a 6‐well plate. After the cells are fully attached to the wells after approximately 10 h, the necessary virus titre is determined using the following formula: virus titre (TU/ml) = MOI value × number of cells/volume of virus (ml). Use an MOI of 10 to infect cells. For subsequent cultures, use lentivirus‐containing medium instead of the initial medium. After 12 h, the medium was replaced with fresh medium and the cells were placed in a thermostat incubator at 37°C. Cells were observed for lentivirus infection under a fluorescence microscope equipped with the appropriate set of filters for GFP detection at 48 h. In addition, lentiviruses include puromycin‐resistant gene sequences, so 2 μg/mL puromycin was used to further minimise the impact of wild‐type cells on subsequent studies.

### 2.2 Cell Counting Kit‐8 (CCK‐8) Assay

The viability of cells was determined using the CCK‐8 test reagent. Cells (3 × 10^3^) in good proliferative activity were inoculated into 96‐well culture plates. Cell proliferation was assessed after 72 h of treatment with drug treatments after apposition. The vitality of the cells was evaluated using an absorbance measurement at 450 nm. The IC50 was determined using the formula. The Chou‐Talalay method was used to evaluate the synergistic impact of the treatments. Combo score values of < 1, = 1, and > 1 showed an antagonistic relationship, additive impact and synergistic relationship, respectively. We performed the selection of experimental drug concentrations through SynergyFinder. The inhibition fraction data of different concentrations of AA and DHA were entered into SynergyFinder for calculation, and the results showed that the combination of the two had a synergistic effect, and the lowest concentration that exerted the greatest effect inside the A549 and H1299 cells was selected.

### 2.3 Colony Formation Assay

H1299 and A549 cells (8 × 10^2^) were seeded into 6‐well plates. Following cell adhesion, the concentration of AA and DHA selected in the CCK‐8 experiment (2 mmol/L of AA and 6.25 μmol/L of DHA) was applied for 24 h. The cells underwent a 2‐week growth phase at 37°C in an incubator. After the cell colonies became visible, the cells were fixed with methanol for 20 min and incubated with crystal violet. Colony images were taken, and the relative colony number of the treatment group (%) was calculated based on the control group.

### 2.4 Flow Cytometry

Cells from drug treatment and control groups were collected and then detected using the Annexin V‐FITC/PI Apoptosis Detection Kit. First, 100 ~ 150 μL of 1 × Binding Buffer working solution was added to resuspend the cells, 100 μL of cell suspension was aspirated into a new tube, and 5 μL of Annexin V‐FITC and 5 μL of PI were added and gently mixed. The cells were incubated at room temperature and protected from light for 15 min. Next, 400 μL of 1 × Binding Buffer was added to each tube and gently mixed, and a single cell suspension was prepared by filtering the cell suspension through nylon cloth into a flow‐through tube. Finally, 10,000 cells from each sample were detected using the BD FACSCalibur flow‐through instrument.

### 2.5 Transwell Migration and Invasion Assays

Cell migration was assessed using a transwell chamber containing 24 wells, whereas invasion was detected in a transwell chamber covered with matrigel. Transwell chambers (24‐well, 8‐mm wells) were placed into empty 24‐well plates, and cells were digested and resuspended in serum‐free medium for counting. Next, 100 μL of cell suspension (3 × 10^4^ cells) was added to the upper chamber of the transwell, while RPMI‐1640 medium containing 20% fetal bovine serum was added to the lower chamber and incubated at 37°C for 24 h. Cells adhering to the membranes were fixed in 4% paraformaldehyde for 30 min and stained with 0.1% crystal violet for 20 min. Finally, the membranes were washed and dried for microscopic counting and analysis.

### 2.6 Determination of Intracellular ROS

After collecting the cells, 1 mL of serum‐free culture medium containing 1 μL of DCFH‐DA (1:1000 dilution to make the concentration of 10 μmol/L) was added to the cells and incubated for 20 min at 37°C. Next, 1 μL of the positive control (Rosup) was added and incubated for 20 min. Reverse mixing was performed every 3–5 min to ensure full contact between the antibody and cells. After washing the cells, 400 μL serum‐free culture medium was added, the cells were mixed, and red fluorescence was detected at the excitation wavelength of 525 nm with a flow cytometer (FACSCanto11). In addition, the light scattering was detected.

### 2.7 Fe^2+^ Assay

We detected divalent iron levels in each group of A549 and H1299 cells by applying the iron assay kit (bc5415, Solarbio, China) per the instructions in the operating manual.

### 2.8 GSH Assay

The reduction in GSH levels in treated A549 and H1299 cells was measured using a GSH kit (E2015, Applygen, China) per the reference protocol provided by the manufacturer.

### 2.9 MDA Assay

The MDA assay kit (E2019, Applygen, China) was used to measure the specific MDA content in the supernatants of treated cells.

### 2.10 Measurement of Mitochondrial Membrane Potential (MMP)

Using the JC‐1 dye kit, the MMP was measured per the manufacturer's recommendations (C2006; Beyotime).

### 2.11 Fluorescence In Situ Hybridization (FISH)

GenePharma (Shanghai, China) designed and synthesised the fluorescence‐tagged GPX4 probe and the cysteine‐labelled SLC7A11 probe. The probe sequences were as follows: SLC7A11‐Homo FISH Probe 5′‐TTATGAGGAGTTCCACCCAGAC‐3′; GPX4‐Homo FISH Probe 5′‐TTTACTTCGGTCTTGCCTCACT‐3′. H1299 and A549 cells were placed on coverslips, then fixed, permeabilized in phosphate‐buffered saline (PBS) containing 0.5% Triton X‐100, and dehydrated in ethanol. The FISH probes were diluted, denatured, and equilibrated before adding to the cells for hybridization overnight at 37°C. After hybridization, the cells were stained with 4′,6‐diamidino‐2‐phenylindole (DAPI) at room temperature. The slides were sealed with rubberized adhesive and placed in the dark for more than 20 min, and images were taken by fluorescence microscopy.

### 2.12 Real‐Time Polymerase Chain Reaction (PCR)

Total RNA was isolated using TRIzol. SuperScript III was used to perform reverse transcription of the extracted RNA into cDNA. Real‐time PCR was subsequently employed to quantify the mRNA expression levels of specific genes. The primer sequences are provided below:

Human *SLC7A11* forward: 5′‐GGTCAGAAAGCCTGTTGT‐3′,

Human *SLC7A11* reverse: 5′‐GTTCCACCCAGACTCGTA‐3′,

Human *GPX4* forward: 5′‐CCTTTGCCGCCTACTGAA‐3′,

Human *GPX4* reverse: 5′‐ACTCCCTGGCTCCTGCTT‐3′,

Human *β‐actin* forward: 5′‐CCTGGCACCCAGCACAAT‐3′,

Human *β‐actin* reverse: 5′‐GCCGATCCACACGGAGTACT‐3′,

mouse *Slc7a11* forward: 5′‐GGCACCGTCATCGGATCAG‐3′,

mouse *Slc7a11* reverse: 5′‐CTCCACAGGCAGACCAGAAAA‐3′,

mouse *Gpx4* forward: 5′‐GCCTGGATAAGTACAGGGGTT‐3′

mouse *Gpx4* reverse: 5′‐CATGCAGATCGACTAGCTGAG‐3′,

mouse *β‐actin* reverse: 5′‐TTTGGGGGATGTTTGCTCCA‐3′,

mouse *β‐actin* forward: 5′‐TGAGCTGCGTTTTACACCCT‐3′.

### 2.13 Western Blot

RIPA lysis buffer containing protease inhibitors (RIPA:phenylmethylsulfonyl fluoride (PMSF) = 100:1) was used to extract the proteins from LUAD cells. The protein concentration was estimated through the biochromatic assay (BCA) kit. The proteins (40 μg/lane) were separated using 10% or 12% sodium dodecyl‐sulfate polyacrylamide gel electrophoresis (SDS‐PAGE) and then transferred onto polyvinylidene difluoride (PVDF) membranes. After blocking in skimmed experimental milk (5%) for 2 h at 37°C, the membranes were incubated with the following primary antibodies: anti‐SLC7A11 (ab300667; 1:1000; Abcam; USA), anti‐SLC7A11 (56243–1; 1:1000; SAB; USA), and anti‐GPX4 (67763–1‐Ig; 1:2500; Proteintech; China). The membranes were incubated in the appropriate secondary antibody the next day. Finally, an enhanced chemiluminescence (ECL) kit was used to visualise the protein signals on the finished membrane (Abbkine, China). Quantitative analysis of western blotting bands was performed using ImageJ software, and the grey values of the internal reference proteins were normalised for grey value analysis.

### 2.14 Animal Experiments

The Kunming Medical University Experimental Animal Committee granted consent for the study, which was conducted at Yunnan Tumour Hospital (approval number: kmmu20221681, address: Ethics Office, Yunnan Cancer Hospital). Female BALB/c mice aged 6–8 weeks underwent subcutaneous injection of LLC cells (2.5 × 10^6^). In this experiment, mice were housed in standard cages at 20°C–24°C with a 12/12‐h light–dark cycle and free access to food and water. Once the tumours grew to a diameter of 6 mm, the mice were randomly allocated into five groups (*n* = 5 per group) and treated as follows: 4 g/kg AA (AA group), 12.5 mg/kg DHA (DHA group), 4 g/kg AA plus 12.5 mg/kg DHA (combined treatment group), and PBS (control), combined with dipeptidyl peptidase‐4 DDP (positive control for chemotherapy, 2 mg/kg), intraperitoneally once a day for 1 week. Mice were monitored every 2 days for body weight and tumour volume. On Day 21, all mice were euthanized by intraperitoneal injection of sodium pentobarbital (150 mg/kg), followed by cervical dislocation as a second method of euthanasia, and death was determined after the mice were immobile, did not breathe, and had dilated pupils, and tumour tissue was collected and imaged. The humane experimental endpoints included tumour weight being > 10% of animal body weight, tumour volume exceeding 2000 mm^3^, and weight loss of > 20% animal body weight; no animals reached these criteria during the experiment. The number of mice used and euthanized was 25, and the cause of death in all animals was euthanasia by intraperitoneal injection of sodium pentobarbital. No premature deaths of mice were found. The *q* value was determined as follows: *q* = E_A+B_/ (EA + EB − EA × EB).

EA + B denotes the combination treatment's inhibition rate, whereas EA and EB represent the single treatment's inhibition rate. Combination therapy effects are classified as antagonistic (*q* < 0.85), additive (0.855 < *q* < 1.15), or synergistic (*q* > 1.15).

### 2.15 RNA Sequencing

RNA extraction was performed using an mRNA kit following the recommended protocols for treating cell lines. Once the libraries were set up, the edgeR package produced reads per kilobase of transcript per million mapped reads (RPKM) data. The change in expression test was performed using the limma‐R program. Heatmap creation was performed using minmax data normalisation. The parameters selected for screening differentially expressed genes were |log2 fold‐change (FC)| ≥ 2 and false discovery rate (FDR) ≤ 0.01. We performed transcriptome sequencing through UW Genetics' DNBSEQ platform and data analysis through its online platform Dr. Tom. Dr. Tom's website (https://biosys.bgi.com/) was used for the Kyoto Encyclopedia of Genes and Genomes (KEGG) pathway analysis of the data. In RNA sequencing, we sequenced 12 samples using the BGISEQ platform, averagely generating about 6.69G Gb bases per sample. The average mapping ratio with the reference genome is 96.86%, the average mapping ratio with genes is 81.19%, and 17,012 genes were identified. SOAPdenovo, developed by BGI, performs genome assembly by utilising a method for assembling short reads, which uses kmer as the node unit and the de Brujin graph approach to achieve whole genome assembly. In order to reflect the correlation of gene expression between samples, the Pearson correlation coefficients of all gene expressions between each two samples were calculated, and these coefficients were reflected in the form of a heatmap. The correlation coefficients can reflect the similar situation of the overall gene expression between each sample. The higher the correlation coefficient is, the more similar the gene expression level is. The sequencing length is PE150. The raw data contains reads of low quality, reads with adaptor sequences and reads with high levels of N base. Those reads need to be filtered before the data analysis for reliability analysis results. We used HISAT to align the clean reads to the reference genome, and used Bowtie2 to align the clean reads to the reference genes. The reference genome version is GCF_000001405.39_GRCh38.p13.

### 2.16 Statistical Analysis

The data were expressed as the mean and standard error of the mean (SEM). GraphPad Prism was used to perform normality tests. Comparisons of continuous variables with a normal distribution were performed using the unpaired Student's *t*‐test for two groups. In contrast, variables with a skewed distribution and ordinal data were compared with the Mann–Whitney *U* test for two groups and the Kruskal–Wallis test for more than two groups. The post hoc tests we used were Sidak's Test and Dunnett's Test. Data were statistically evaluated using GraphPad Prism 8.0.2.

## 3 Results

### 3.1 AA Combined With DHA Inhibited LUAD Cell Proliferation

**FIGURE 1:**
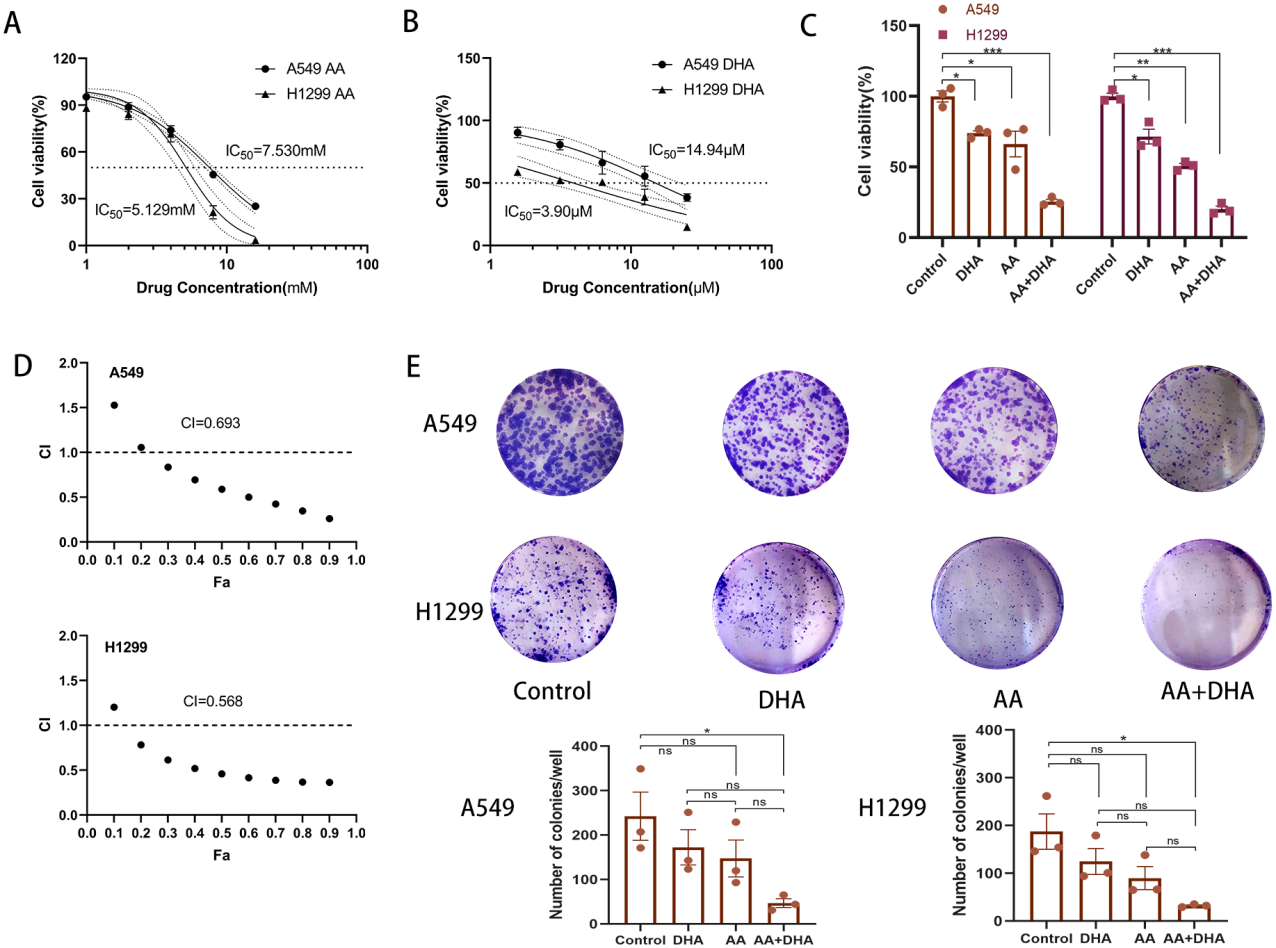
In vitro lung adenocarcinoma (LUAD) cell growth was reduced by the combination of ascorbic acid (AA) and dihydroartemisinin (DHA). A549 and H1299 cells were treated with different doses of AA (0–32 mmol/L) (A) or DHA (0–100 μmol/L) (B) for 72 h, and cell viability was determined by the CCK‐8 assay. (C) Comparison of cell viability in A549 and H1299 cells treated with AA and DHA for 72 h. (D) Combination index (CI) plots showing the anticancer activity of AA combined with DHA at different concentrations in A549 and H1299 cell lines. CI values are shown against fractional growth inhibition (Fa). The CI values for antagonistic behaviour, additivity, and synergy are > 1, = 1, and < 1. (E) In A549 and H1299 cells, the combination of AA and DHA inhibited the growth of cell colonies. *p < 0.05, **p < 0.01, ***p < 0.001.

A549 and H1299 cells were treated with different doses of AA (ranging from 0 to 32 mmol/L) or DHA (ranging from 0 to 100 μmol/L) for 72 h. The CCK‐8 assay showed that both AA and DHA suppressed cell growth in a concentration‐dependent manner. Furthermore, the combined treatment had a more pronounced inhibitory effect than each treatment alone. The IC50 value of the two cell lines differed for each medication; AA had IC50 values of 5.129 mmol/L in H1299 cells and 7.530 mmol/Lin A549 cells, and DHA had IC50 values of 14.94 μmol/L for H1299 cells and 3.90 μmol/L for A549 cells (Figure 1A,B). Data on the inhibition scores of the different concentrations of AA and DHA were entered into SynergyFinder, which showed that the combination of the two treatments had a synergistic effect. The most efficient combination for suppressing cell activity was 2 mmol/L of AA and 6.25 μmol/L of DHA (Figure S1), which led to a decrease of 20.20% in H1299 cells and 25.85% in A549 cells (*p* < 0.001) (Figure 1C). Additionally, additional combinatorial index (CI) calculations revealed that AA and DHA worked in concert (CI < 1) to prevent the growth of cancer cells (Figure 1D). In addition, following treatment with AA combined with DHA for 12 days, colony formation in the A549 and H1299 cells was significantly reduced compared to that in the monotherapy groups (*p* < 0.05) (Figure 1E).

### 3.2 AA Combined With DHA Prevented LUAD Cell Migration and Invasion

**FIGURE 2:**
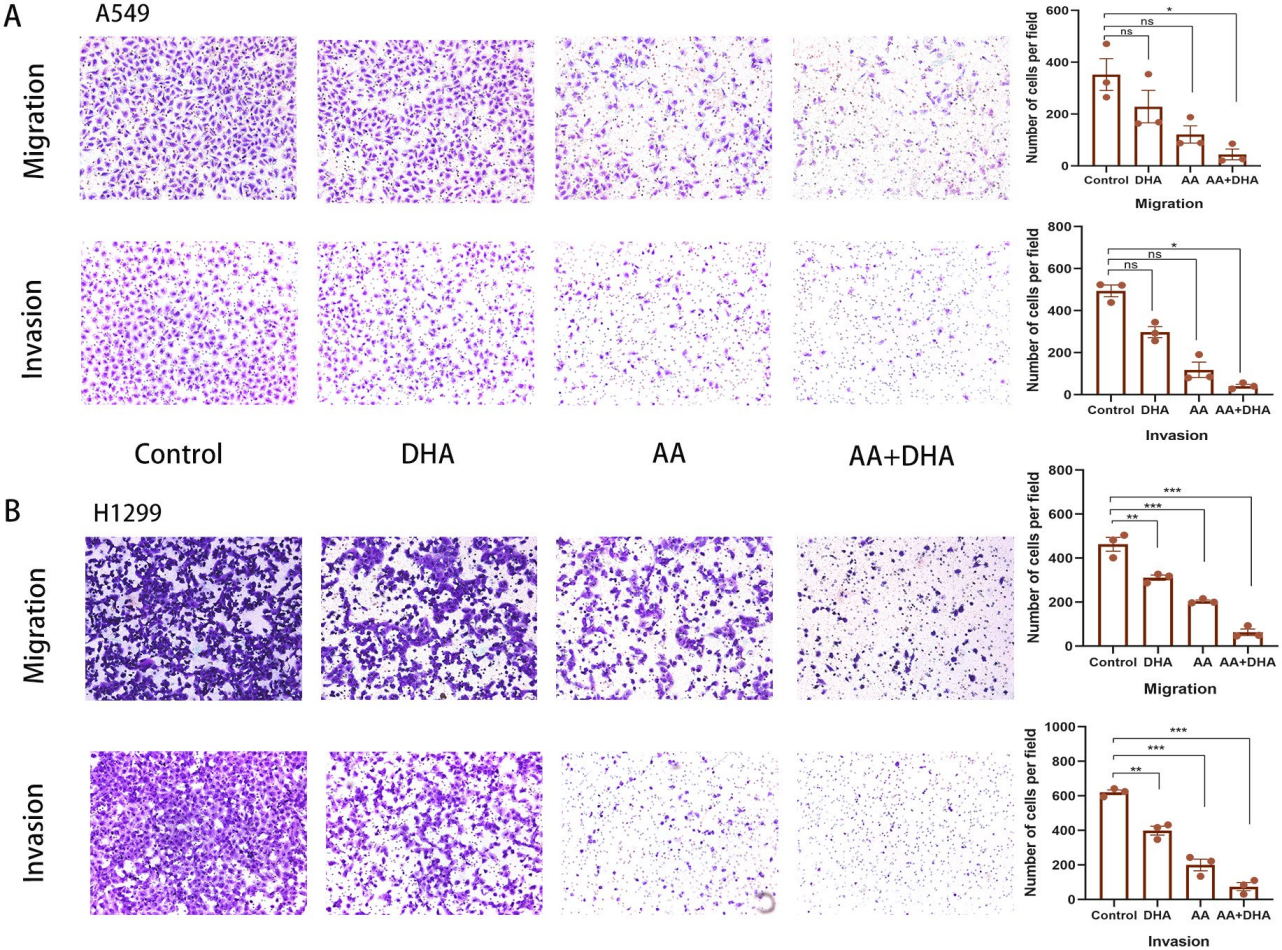
The combination of ascorbic acid (AA) and dihydroartemisinin (DHA) prevented lung adenocarcinoma (LUAD) cells from migrating and invading in vitro. (A, B) The transwell assay was used to examine migration and invasion in A549 and cells following treatment. *p < 0.05, **p < 0.01, ***p < 0.001.

The impact of combining AA and DHA on the migration and invasion of LUAD cells was evaluated using the transwell assay. The results showed that AA and DHA significantly reduced the migration of A549 (*p* < 0.05) (Figure 2A) and H1299 (*p* < 0.001) (Figure 2B) cells. Furthermore, following treatment with AA and DHA, the invasion capacity of LUAD cells was markedly reduced. These findings demonstrate that AA and DHA prevented LUAD cell migration and invasion.

### 3.3 AA Combined With DHA Increased LUAD Cell Apoptosis

**FIGURE 3:**
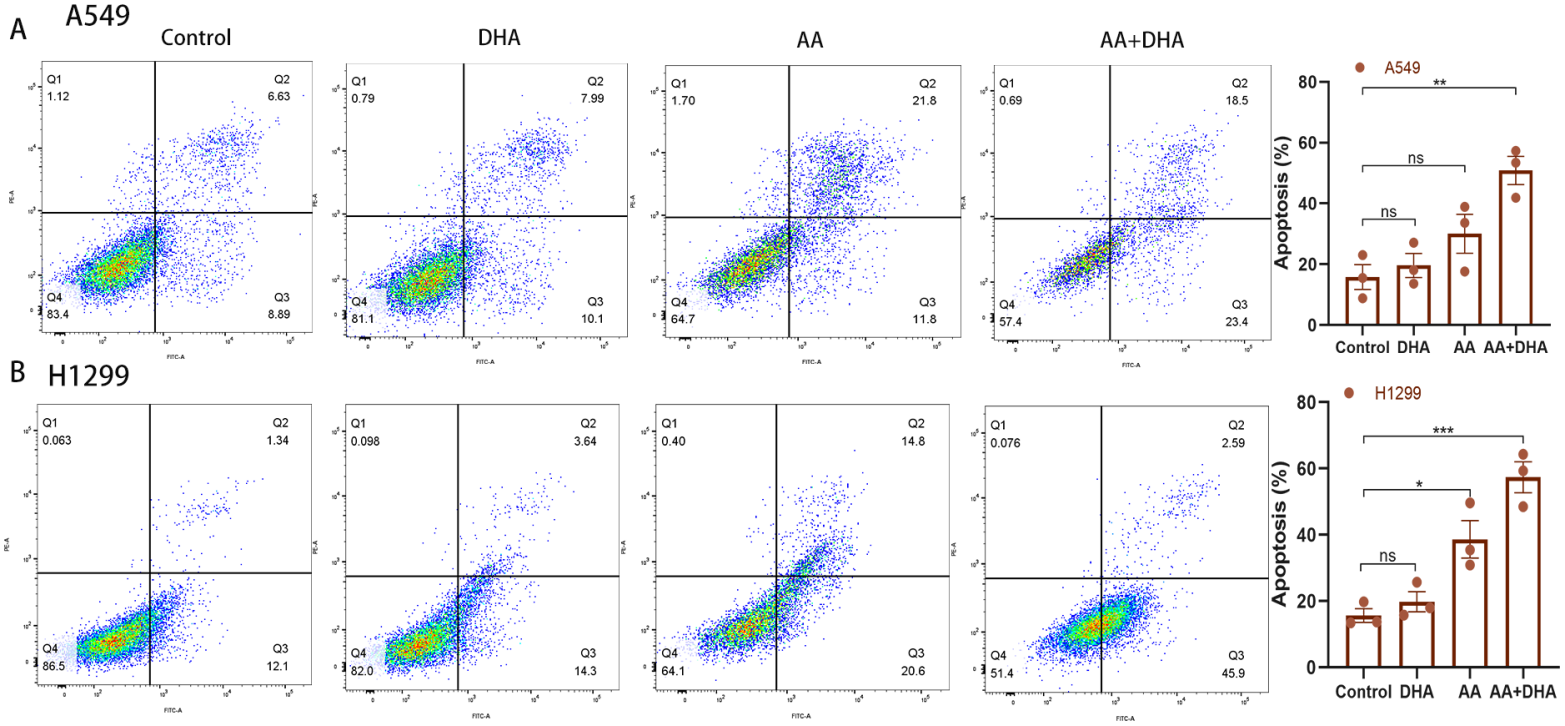
In lung adenocarcinoma (LUAD) cells, ascorbic acid (AA) and dihydroartemisinin (DHA) caused apoptosis. (A, B) An Annexin V‐FITC/PI staining assay was used to measure the degree of cellular apoptosis, and the corresponding percentage of apoptotic cells was computed. *p < 0.05, **p < 0.01, ***p < 0.001, ****p < 0.0001.

To verify the apoptosis‐inducing role of AA combined with DHA in lung cancer, A549 and H1299 cells were treated with AA, DHA and AA plus DHA for 72 h. The results showed that the combined treatment significantly increased the number of apoptotic cells in both A549 and H1299 cells compared to the control and individual drug groups. The apoptosis ratio in A549 cells exhibited an increase from 15.79% (control), 19.61% (DHA), and 30.04% (AA) to 50.87% (AA combined with DHA), as shown in Figure 3A (*p* < 0.01). Similarly, in H1299 cells, the apoptotic proportion increased from 15.64% (control), 19.80% (DHA), and 38.63% (AA) to 57.36% (AA combined with DHA), as shown in Figure 3B (*p* < 0.001).

### 3.4 AA Combined With DHA Reduced the Progression of LLC Tumours In Vivo

**FIGURE 4:**
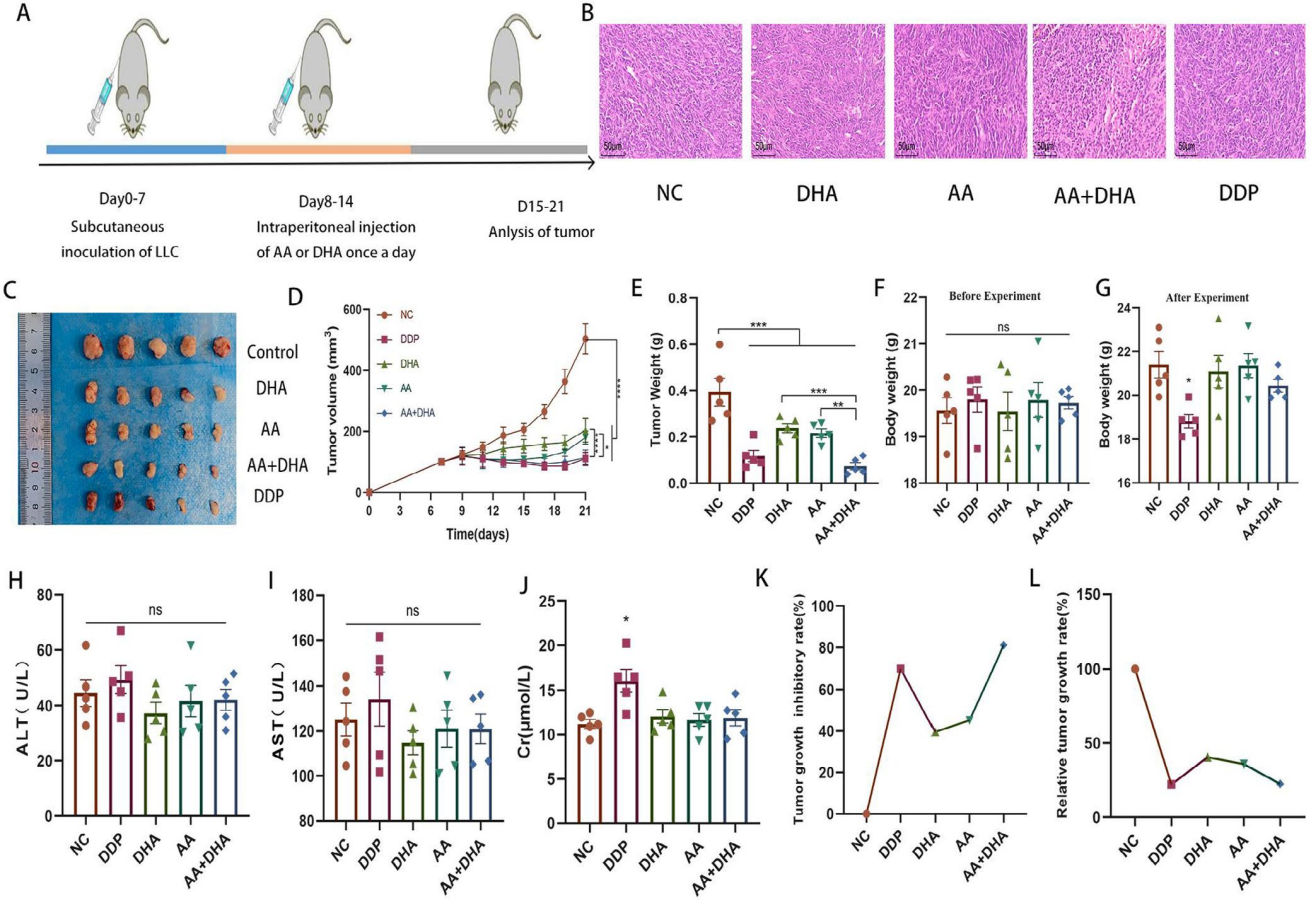
AA combined with DHA reduced the progression of LLC tumours in vivo. (A) A schematic representation of the animal experimentation procedure. (B, C, D, E) Haematoxylin and eosin staining, images, weight statistics, and tumour growth curves analysis of tumours excised from mice in the control, AA, DHA, AA combined with DHA and DDP groups. (F, G) Body weights of LLC mice in each group before and after the experiment. (H, I, J) ALT, AST, and Cr levels of mice in control, AA, DHA, AA combined with DHA and DDP groups. (K, L) Line graphs of tumour growth inhibition and relative growth rates in control, AA, DHA, AA combined with DHA and DDP groups. DDP is used as a positive control for chemotherapy. *p < 0.05, **p < 0.01, ***p < 0.001.

To evaluate the antitumor efficacy of AA combined with DHA in vivo, we established LLC tumour models in BALB/c mice (Figure 4A). The mice were sacrificed on Day 21, and the tumours were extracted. Haematoxylin and eosin (HE) staining showed that tumours did form subcutaneously in the mice (Figure 4B). Figure 4C–E demonstrates that tumours treated with AA and DHA had a significant reduction in the average size (*p* < 0.001) and weight (*p* < 0.001) compared to the control group. The body weights and liver and kidney functions of the mice before and after the experiment were observed, and there were no significant changes in body weight, alanine aminotransferase (ALT), aspartate aminotransferase (AST), and creatinine (Cr) in the other groups, except for the weight loss and elevated kidney function in the positive control group after the test (*p* > 0.05) (Figure 4F–J). HE staining was used to study the toxicity of the combination treatment on major organs. The results showed no significant abnormalities or obvious organ damage in the heart, liver, spleen, lung, kidney, and brain organ (Figure S2). The tumour inhibition rate and relative tumour growth rate of each group were counted, and the results showed that the combined group significantly inhibited the growth of tumours in vivo (Figure 4K,L). The *q* for AA plus DHA was 1.21, indicating synergism in the combination group, and the combination of AA and DHA did not affect the body weight, liver and kidney functions, and vital organs of the mice, which suggests that both AA and DHA inhibit tumours in mice without exhibiting any significant deleterious effects.

### 3.5 AA Combined With DHA‐Induced Tumour Cell Ferroptosis

**FIGURE 5:**
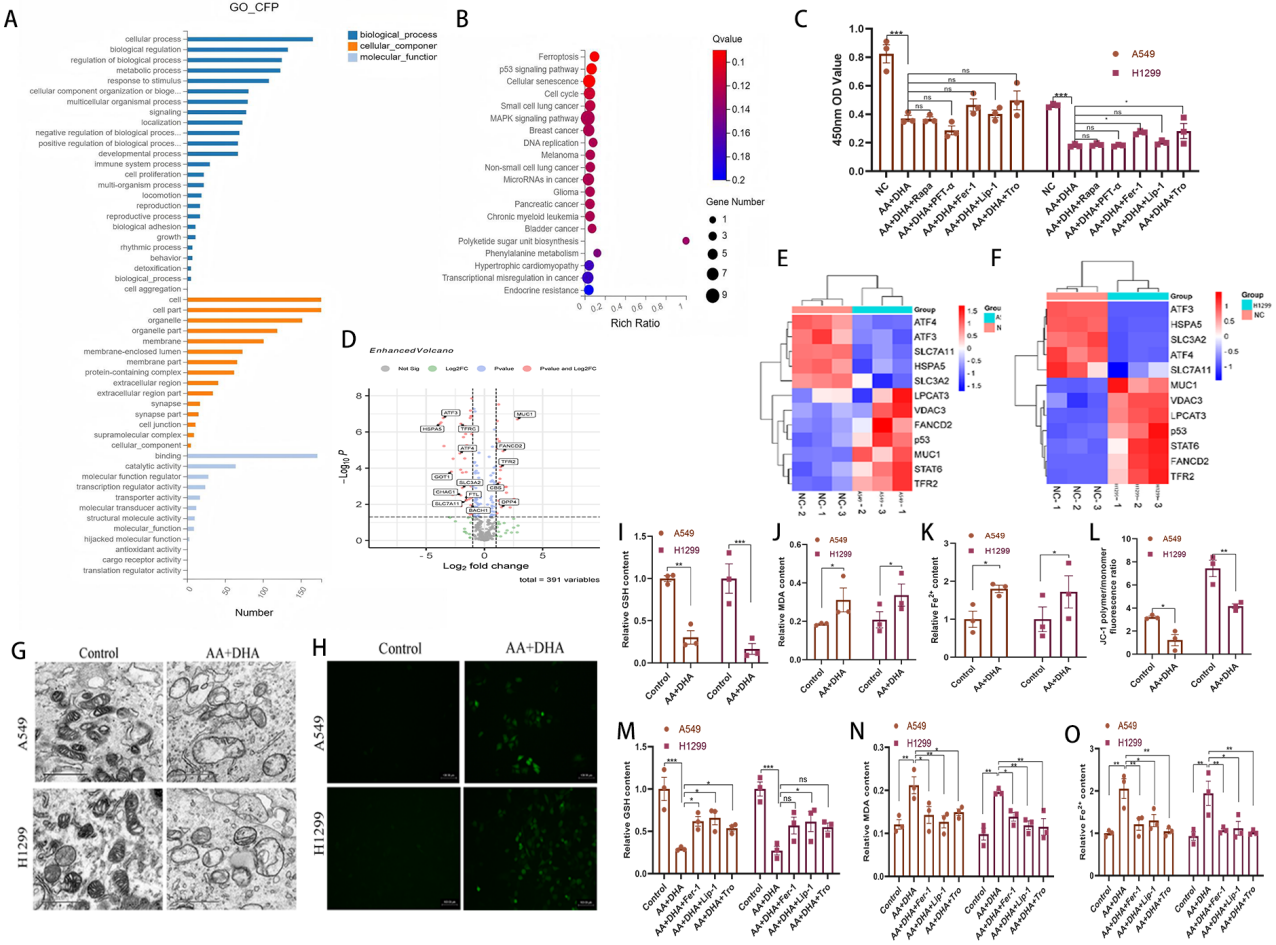
Induction of tumour cell ferroptosis by ascorbic acid (AA) combined with dihydroartemisinin (DHA). (A) Gene Ontology (GO) and histograms analysed differential genes and plotted them against targets selected for scoring. (B) The differential genes were subjected to Kyoto Encyclopedia of Genes and Genomes (KEGG) analysis, and the top 20 representative signalling pathways were selected for bubble mapping based on the scores. (C) The CCK‐8 assay was used to assess the proliferation of A549 and H1299 cells induced by AA combined with DHA following treatment with Rapa, PFT‐a, Fer‐1, Lip‐1, and Tro. (D, E, F) Volcano and heat maps of genes screened by RNA sequencing compared to the FerrDb show the differential expressions of ferroptosis genes. (G) Transmission electron microscopy was used to investigate the ultrastructure of A549 and H1299 cells. The reactive oxygen species (ROS) (H), glutathione (GSH) (I), malondialdehyde (MDA) (J), iron content (K), and JC‐1 (L) were measured by the corresponding kit. (M, N, O) GSH, MDA, and iron content levels in A549 and H1299 cells were detected after AA plus DHA treatment with or without Fer‐1, Lip‐1 and Tro. *p < 0.05, **p < 0.01, ***p < 0.001.

We next sought to define the molecular mechanism by which AA combined with DHA prevented tumour progression. We first performed unbiased RNA sequencing in A549 and H1299 cells following treatment with AA combined with DHA. We validated the RNA transcriptome sequencing results (Figure S3). The expression of *SLC7A11, AKT, SAT1* and *GDF15* analysed by cellular PCR showed a consistent trend with the sequencing results. Ferroptosis, the P53 signalling pathway, and cellular senescence were the top three representative signalling pathways identified in the KEGG enrichment analysis (Figure 5A,B). Notably, one of the signalling pathways that was considerably enriched was ferroptosis. To functionally evaluate their relative contributions, we performed rescue experiments using specific inhibitors against each. CCK‐8 assays confirmed that while inhibitors of three pathways could partially reverse AA+DHA‐induced cell death, the protective effect of ferroptosis inhibitors (Fer‐1, Lip‐1, Tro) was the most pronounced, as quantitatively demonstrated in Figure 5C. This functional evidence led us to prioritise ferroptosis for in‐depth mechanistic study, interpreting it as the most prominently activated death mode under our experimental conditions. We compared the genes screened by RNA sequencing with the FerrDb (http://www.zhounan.org/ferrdb), and volcano and heat maps showed that ferroptosis genes were differentially expressed (Figure 5D–F). Furthermore, consistent with previously published findings, *SLC7A11* was strongly regulated [8, 24]. We screened *SLC7A11* as a subsequent essential gene and *GPX4* as a downstream pathway.

To demonstrate AA plus DHA‐induced ferroptosis in cancer cells, we investigated the morphology of the treated cells. TEM showed that A549 and H1299 cells demonstrated shrinking mitochondria, a greater density of the mitochondrial membrane, and a decrease in or absence of mitochondrial cristae, which are the physical characteristics of ferroptosis (Figure 5G). It is critical to understand that the process of ferroptosis involves the buildup of MDA, Fe^2+^, and ROS in addition to the reduction of GSH and a decrease in the MMP. As expected, a decreased GSH level (*p* < 0.01) and increased MDA (*p* < 0.05), Fe^2+^ (*p* < 0.05), and ROS levels were observed in the AA plus DHA treatment group (Figure 5H–K). In contrast to the placebo group, the JC‐1 of A549 and H1299 cells was lower after receiving AA and DHA therapy (Figure 5L) (*p* < 0.05). LUAD cells were then treated with the Lip‐1, Fer‐1, and Tro ferroptosis inhibitors (Figure 5M–O). The GSH, MDA, and Fe^2+^assay results indicated that the ferroptosis inhibitors could partly rescue the effects of AA combined with DHA. These results revealed that ferroptosis may be the critical mechanism by which AA plus DHA triggered cell death in LUAD cells.

### 3.6 AA Combined With DHA Induces Ferroptosis in LUAD Cells via the SLC7A11/GPX4 Axis

**FIGURE 6:**
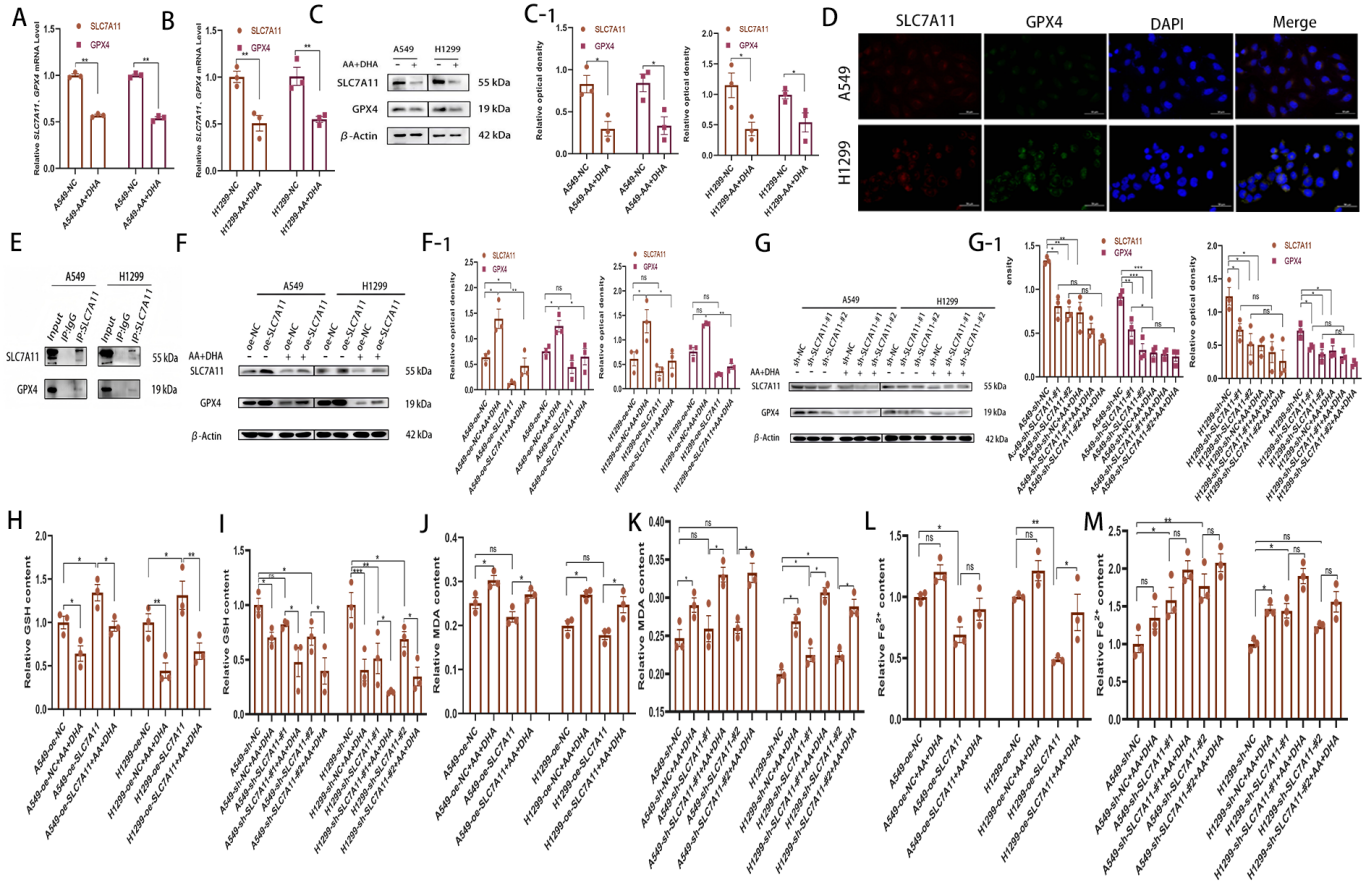
Ascorbic acid (AA) combined with dihydroartemisinin (DHA) induced ferroptosis in LUAD cells via the SLC7A11/GPX4 axis. (A, B) mRNA expression of *SLC7A11* and *GPX4* in A549 and H1299 cells with or without AA plus DHA treatment. (C) Western blotting detected SLC7A11 and GPX4 levels in A549 and H1299 cells with or without AA combined with DHA treatment. (D) Fluorescence in situ hybridization (FISH) confirmed that *SLC7A11* and *GPX4* were mostly located in the nucleus and cytoplasm. (E) Co‐IP results for A549 and H1299 cells. (F) Western blotting detected SLC7A11 and GPX4 levels in A549 and H1299 cells after AA plus DHA treatment with or without oe‐*SLC7A11*. (G) Western blotting detected SLC7A11 and GPX4 levels in A549 and H1299 cells after AA plus DHA therapy with or without sh‐*SLC7A11*. (H, J, L) glutathione (GSH), malondialdehyde (MDA), and Fe^2+^ levels in A549 and H1299 cells were detected after AA plus DHA treatment with or without oe‐*SLC7A11*. (I, K, M) GSH, MDA, and Fe^2+^ levels in A549 and H1299 cells were detected after AA plus DHA treatment with or without sh‐*SLC7A11*. *p < 0.05, **p < 0.01, ***p < 0.001.

To investigate the potential molecular mechanisms of AA combined with DHA‐induced ferroptosis, we selected two pivotal signalling proteins from the ferroptosis pathway, SLC7A11 and GPX4, as the focus of our subsequent research. Treatment with AA combined with DHA was found to significantly reduce the mRNA and protein expression of *SLC7A11* and *GPX4* in LUAD cells compared with the control group (Figure 6A–C). The subcellular location of *GPX4* and *SLC7A11* was investigated using FISH. We found that *SLC7A11* and *GPX4* were enriched in the cytoplasmic and nuclear fractions and were predominantly distributed in the cytoplasm (Figure 6D). Co‐IP experiments were carried out to verify whether SLC7A11 associates with GPX4, and the results showed that SLC7A11 interacts with GPX4 (Figure 6E), which further validates our pathway SLC7A11/GPX4. We further transfected LUAD cells with a lentiviral plasmid overexpressing *SLC7A11* (oe‐*SLC7A11*) or sh‐*SLC7A11* before treatment to confirm whether AA combined with DHA‐induced ferroptosis occurred through *SLC7A11*. The upregulation of *SLC7A11* partially mitigated the AA combined with DHA‐induced decrease in the GPX4 protein levels and GSH content (Figure 6F,H). Furthermore, sh‐*SLC7A11* reduced the AA combined with the DHA‐induced effect on the GPX4 protein level and GSH content in A549 and H1299 cells (Figure 6G,I). In addition, oe‐*SLC7A11* significantly attenuated AA combined with DHA‐induced MDA and Fe^2+^ accumulation; conversely, sh‐*SLC7A11* increased MDA and Fe^2+^ accumulation (Figure 6J–M).

Furthermore, we performed additional validation through GPX4 overexpression. Compared with the control group, GPX4 overexpression did not affect the expression of upstream SLC7A11. However, combined AA and DHA treatment reduced the expression of both SLC7A11 and GPX4 in LUAD cells (Figure S4A). To further investigate the functional interaction between GPX4 and SLC7A11, we overexpressed GPX4 in the context of SLC7A11 knockdown, followed by AA/DHA treatment. Similarly, GPX4 overexpression did not affect upstream SLC7A11 expression, and AA combined with DHA treatment again reduced the expression of both SLC7A11 and GPX4 (Figure S4B). GPX4 overexpression also influenced ferroptosis‐related indicators. Overexpression of GPX4 inhibited ferroptosis, as evidenced by a significant decrease in the level of MDA, the end product of lipid peroxidation, and an indirect reduction in Fe^2+^ level, while GSH showed no significant change. Subsequent treatment with AA combined with DHA induced ferroptosis (Figure S4C,E,G). When GPX4 was overexpressed in the context of SLC7A11 knockdown followed by AA+DHA treatment, GSH decreased, MDA increased from a suppressed state, and Fe^2+^ levels subsequently rose (Figure S4D,F,H). These results demonstrate that the SLC7A11‐GSH axis is an essential support for GPX4 function, and together they constitute an integrated defence system.

**FIGURE 7:**
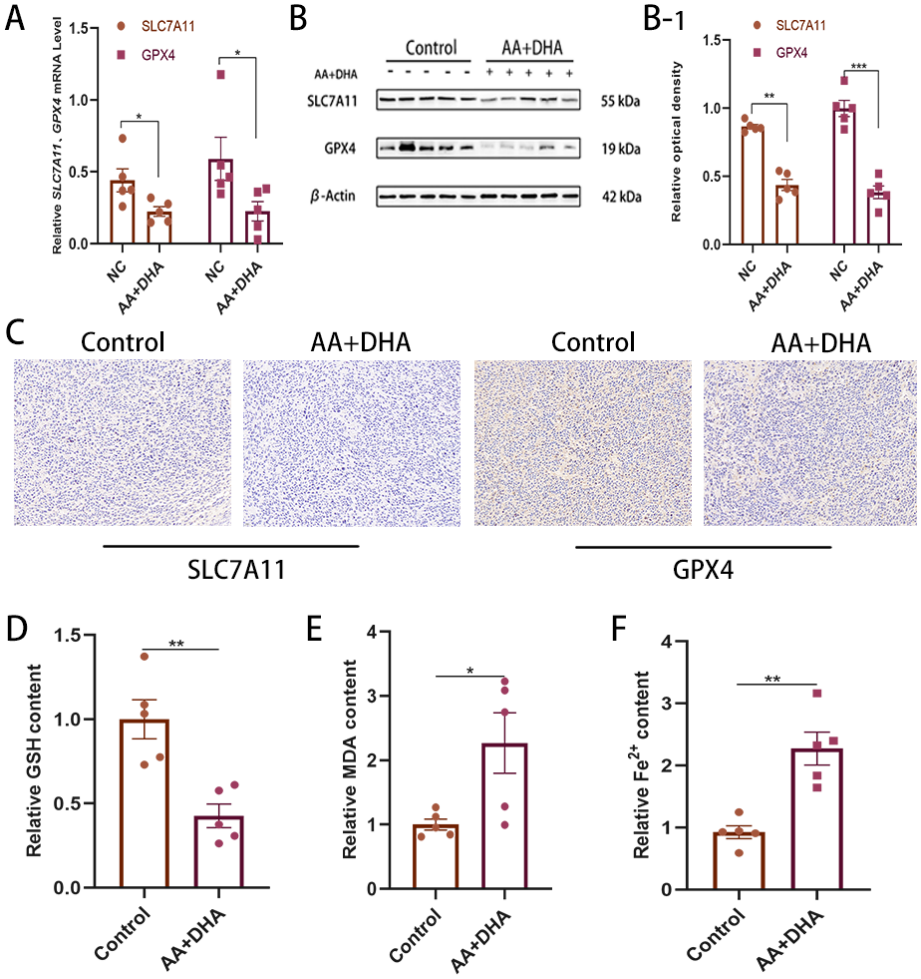
Ascorbic acid (AA) combined with dihydroartemisinin (DHA) induced ferroptosis in LUAD cells via the SLC7A11/GPX4 axis. (A, B) *Slc7a11* and *Gpx4* in tumours from various treatment groups were detected using qPCR and western blotting. (C) Immunohistochemistry determined the expression of Slc7a11 and Gpx4. (D, E, F) Following ascorbic acid (AA) combined with dihydroartemisinin (DHA) treatment, glutathione (GSH), Fe^2+^, and malondialdehyde (MDA) levels were detected in experimental tumour tissues in vivo.*p < 0.05, **p < 0.01, ***p < 0.001 .

To verify whether ferroptosis also occurred in our animal experiments, we examined the mRNA and protein expression of *Slc7a11* and *Gpx4* genes in the tumour tissues. AA combined with DHA decreased the expression of *Slc7a11* and *Gpx4* compared to the control group (Figure 7A,B). Finally, tumour slices were subjected to immunohistochemistry (IHC) to identify protein expression further. Decreased protein levels of Slc7a11 and Gpx4 were found following AA plus DHA treatment (Figure 7C). In addition, we examined indicators of ferroptosis in the experimental tumour tissues. The results showed that combined treatment with AA and DHA decreased the GSH content and elevated the levels of Fe^2+^ and MDA, which was consistent with the results of cellular experiments (Figure 7D–F). Together, AA combined with DHA induced ferroptosis in LUAD cells via regulation of the SLC7A11/GPX4 axis.

## 4 Discussion

LUAD remains a highly prevalent and aggressive malignancy with substantial global morbidity and mortality. Although advances in diagnostics and therapies have modestly improved survival over the past decade, prognosis remains suboptimal for many patients. Key challenges include treatment resistance and drug‐related toxicity, driving the search for complementary approaches. Natural compounds and plant‐derived agents represent promising candidates in this regard, potentially offering reduced side effects while maintaining antitumor efficacy, thereby possibly improving both treatment outcomes and quality of life.

Furthermore, there is broad consensus that combination therapy may offer greater efficacy than monotherapy. Drug resistance emerges due to the dynamic process of tumorigenesis and progression. In addition, given the ineffectiveness of monotherapy, combination treatment offers a practical and efficient way to combat drug resistance [25]. AA and DHA have shown potential as new anticancer agents that selectively target cancer cells [26, 27], and a unique combination therapy using AA and DHA for LUAD has been proposed in this study. The combined toxic effects of these two substances function via the SLC7A11/GPX4 pathway, which may change the aggressive phenotype of LUAD, offering new hope for patients with this disease.

Many studies have shown that AA exhibits different effects depending on the dose, with pharmacological doses of AA having pro‐oxidant properties and physiological doses of AA having antioxidant properties [28]. Large therapeutic doses of AA (1 mM) prevent tumour progression in vivo, mainly via the pro‐oxidant pathway, resulting in tumour cell death in vitro [29]. Pharmacological AA doses induce hydrogen peroxide (H_2_O_2_) production, which reacts with unstable ferrous iron to produce hydroxyl radicals. Hydroxyl radicals can disrupt cell membrane and DNA integrity, alter glucose metabolism, and ultimately lead to cancer cell death [30]. Similarly, DHA has been found to possess antiproliferative, apoptotic cycle‐inhibiting, and ferroptosis‐promoting properties in many tumour cell lines [31, 32]. DHA induces immunogenic cell death in lung cancer by inducing endoplasmic reticulum stress and DNA damage, ultimately inducing cell death [33]. Previous studies have shown that adequate plasma levels, better synergistic effects, and lower toxicity can be achieved using high doses of AA (> 1 g/kg) compared to low doses (< 1 g/kg) [34]. Our animal experiments using an intraperitoneal dose of 4 g/kg showed it to be safe and effective, which is consistent with the findings of synergistic enhancement of therapeutic effect by high doses of AA [35]. We acknowledge that the human equivalent dose (HED) derived from the 4 g/kg AA used in our animal study is considerably higher than conventional clinical doses. However, clinical evidence supports the manageable safety profile of high‐dose intravenous AA in oncology with appropriate precautions. Key risks—hemolysis and oxalate nephropathy—can be mitigated by screening for G6PD deficiency [36] and excluding patients with renal impairment [37]. In patients with normal renal function, less than 0.5% of infused AA (up to 1.5 g/kg) converts to oxalate [38]. Clinical studies, including those using doses up to 1.5 g/kg [39] or 200 g/day [40], have reported no serious adverse events, with only mild side effects such as thirst and polyuria observed in our ongoing trial. Collectively, these findings indicate that the HED in our study falls within a clinically explored and manageable range under medical supervision. Overall, our findings suggest that the combined use of AA and DHA may be a better therapeutic option compared to monotherapy. In addition, AA can inhibit the growth of aggressive malignant tumours vitro by inducing a high accumulation of ROS, which promotes iron death [8, 9, 41]. Similarly, DHA can induce ferroptosis, which provides the basis for our subsequent mechanistic studies. Overall, the combination of AA and DHA consistently affects LUAD malignant behaviour and may be useful in treating patients with LUAD.

Based on our preclinical findings, the combination of ascorbic acid (AA) and dihydroartemisinin (DHA) significantly inhibited LUAD progression both in vitro and in vivo, primarily through induction of ferroptosis. Mechanistically, we demonstrated that the AA‐DHA combination cooperatively disrupts the SLC7A11/GPX4 signalling axis, leading to decreased glutathione (GSH) levels, increased lipid peroxidation, Fe^2+^ accumulation, and mitochondrial membrane potential reduction—collectively confirming ferroptotic cell death. These results align with existing literature reporting the ferroptosis‐inducing potential of both compounds. Previous studies indicate that DHA promotes ferroptosis via the PRIM2/SLC7A11 axis [42], while AA can trigger GPX4 degradation and support redox cycling with iron [24, 43]. Our study extends these findings by revealing a synergistic interaction between AA and DHA through co‐regulation of the SLC7A11/GPX4 pathway. Additionally, emerging evidence suggests both compounds may modulate the tumour immune microenvironment [44, 45], pointing to a promising direction for future research into combining this regimen with immunotherapy. While these preclinical results highlight a potentially valuable therapeutic strategy, further validation remains necessary to assess its clinical applicability in LUAD treatment.

Thus far, the mechanism by which AA combined with DHA affects solid tumours has not been studied. Our study first explored the efficacy of AA combined with DHA in treating LUAD. We found that it can increase programmed cell death and inhibit LUAD cell migration, infiltration, and growth. This result may have implications for LUAD treatment. It is critical to recognise the limitations of this study. The sample size for the animal experiments in this study was relatively insufficient. Although validated through cellular and animal studies, it is classified as preclinical research. Among the main drawbacks is the absence of validation from prospective clinical trials, which might support the safety and effectiveness of the treatment plan. Currently, the research team is conducting clinical trials with high‐dose AA, aiming to provide clinical evidence for this study and develop treatment options for advanced‐stage patients. Specifically, we did not include direct detection of markers for other cell death pathways, such as Cleaved Caspase‐3 for apoptosis or phosphorylated MLKL for necroptosis. Beyond the programmed cell death pathways we have selected, mechanisms such as the p53 signalling pathway and cellular senescence, along with other potentially undiscovered death pathways, may still exert auxiliary or synergistic effects. Additionally, the SLC7A11/GPX4 pathway might not represent the sole mechanism in this therapeutic approach, and transcriptome sequencing could potentially unveil additional mechanisms. Furthermore, the type and number of cancer cells deserve further investigation, such as adding chemotherapy‐ and targeted therapy‐resistant LUAD cell lines and other cancer cell lines for combinatorial studies to make the findings more convincing. In subsequent studies, these are the directions for the improvements in our research. Despite these limitations, the results of this study offer hope for the development of new, effective treatments for LUAD.

This preclinical study provides preliminary evidence that the AA‐DHA combination suppresses LUAD progression in experimental models, potentially through regulation of the SLC7A11/GPX4 ferroptosis axis. While these findings offer a potential therapeutic strategy worthy of further investigation, their clinical applicability requires validation through subsequent safety and efficacy studies.

## Author Contributions

**Lan Li:** conceptualization (equal), investigation (equal), methodology (equal), resources (equal), software (equal), validation (equal), visualization (equal), writing – original draft (equal). **Fei Lu:** investigation (equal), methodology (equal), software (equal), validation (equal), visualization (equal), writing – original draft (equal). **Sisong Shu:** methodology (equal), software (equal), validation (equal), visualization (equal), writing – original draft (equal). **Xiao Jiang:** investigation (equal), methodology (equal), resources (equal), validation (equal), visualization (equal). **Han Lu:** methodology (equal), software (equal), validation (equal), visualization (equal). **Ke Cao:** investigation (equal), validation (equal), visualization (equal). **Zhengting Chen:** investigation (equal), validation (equal), visualization (equal). **Jingyan Gao:** methodology (equal), validation (equal), visualization (equal). **Mengyuan Liu:** investigation (equal), methodology (equal), resources (equal). **Li Chang:** conceptualization (equal), data curation (equal), project administration (equal), supervision (equal), writing – review and editing (equal). **Wenhui Li:** conceptualization (equal), data curation (equal), formal analysis (equal), funding acquisition (equal), project administration (equal), resources (equal), supervision (equal), writing – review and editing (equal).

## Funding

This study was supported by Yunnan Provincial Department of Education Science Research Fund Project (No. 2022Y220), Yunnan Provincial Training Special Funds for High‐Level Health Technical Personnel (Nos. L‐2018001 and D‐2019030), and Fundamental Research Projects (Nos. 202401AT070014 and 202301AY070001‐240).

## Ethics Statement

*Animal Studies*: All animal studies have been approved by the Experimental Animal Committee of Kunming Medical University (permission number: kmmu20221681). The Yunnan Cancer Hospital's Ethics Office can be found at 519 Kunzhou Road in Kunming, Yunnan Province, China.

## Consent

The authors have nothing to report.

## Conflicts of Interest

The authors declare no conflicts of interest.

## Supporting information


**Data S1:** jcmm70993‐sup‐0001‐Figures.docx.

## Data Availability

All data generated in the present study may be requested from the corresponding author.
