# Supplementary material for: High‐Dose Ascorbic Acid Combined With Dihydroartemisinin Inhibits Lung Adenocarcinoma Malignancy by Inducing Ferroptosis via SLC7A11/GPX4 Pathway
Source: J Cell Mol Med. 2025 Dec 26;29(24):e70993. doi: 10.1111/jcmm.70993 (PMC12741917; doi:10.1111/jcmm.70993)
Supplement: Supplementary file 1 — Data S1: jcmm70993‐sup‐0001‐Figures.docx. [file JCMM-29-e70993-s001.docx]

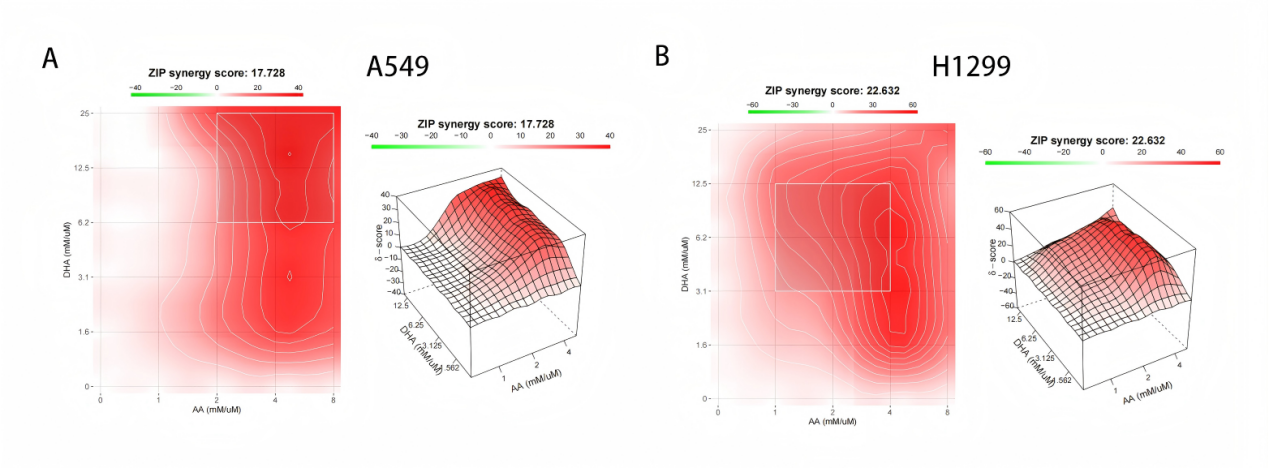


**Supplementary Figure 1.** (A, B) Calculation of the synergistic effect of AA combined with DHA in A549 and H1299 cells using SynergyFinder.


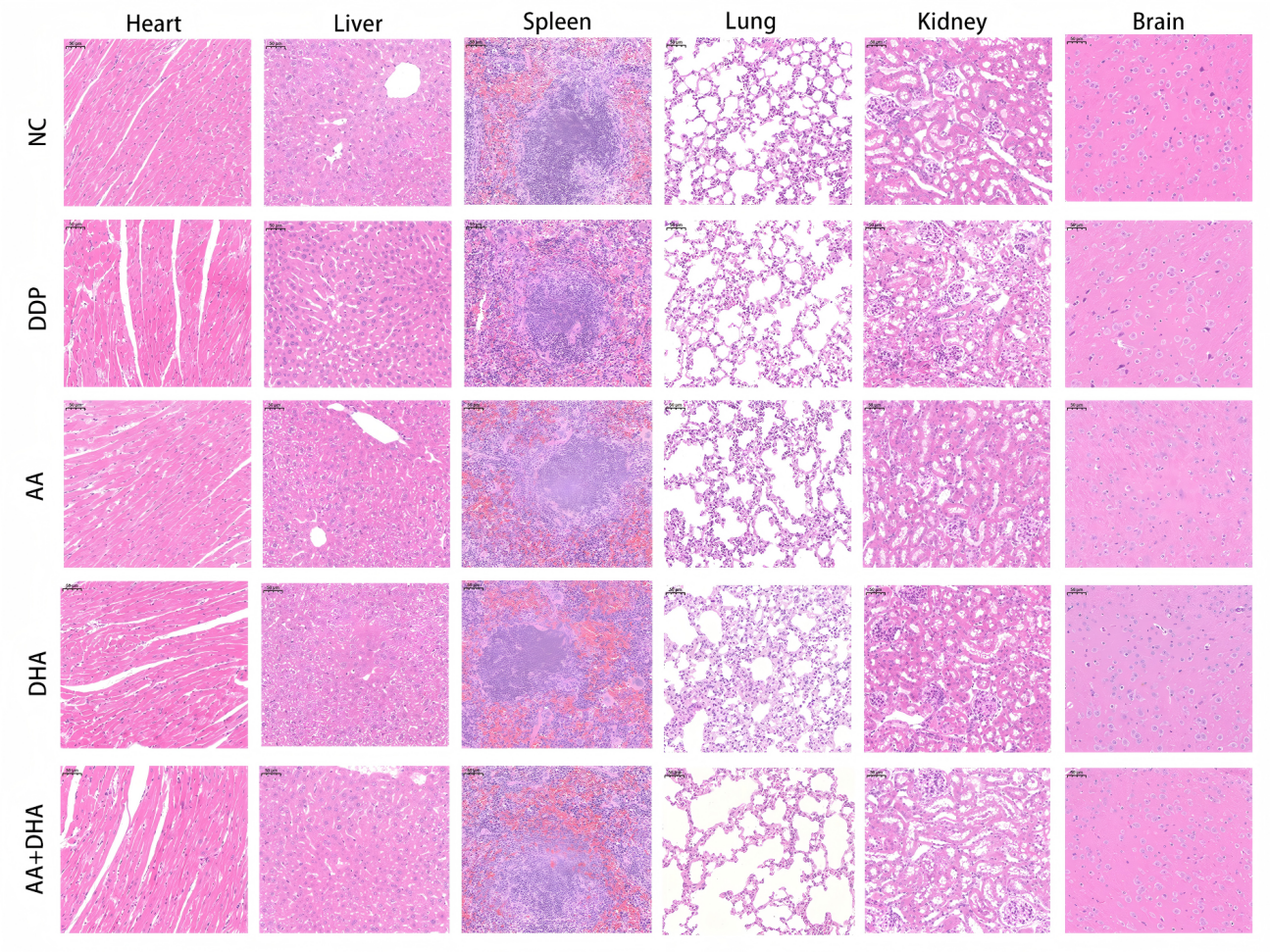


**Supplementary Figure 2.** Photographs of HE-stained sections of heart, liver, spleen, lung, kidney and brain of each group.


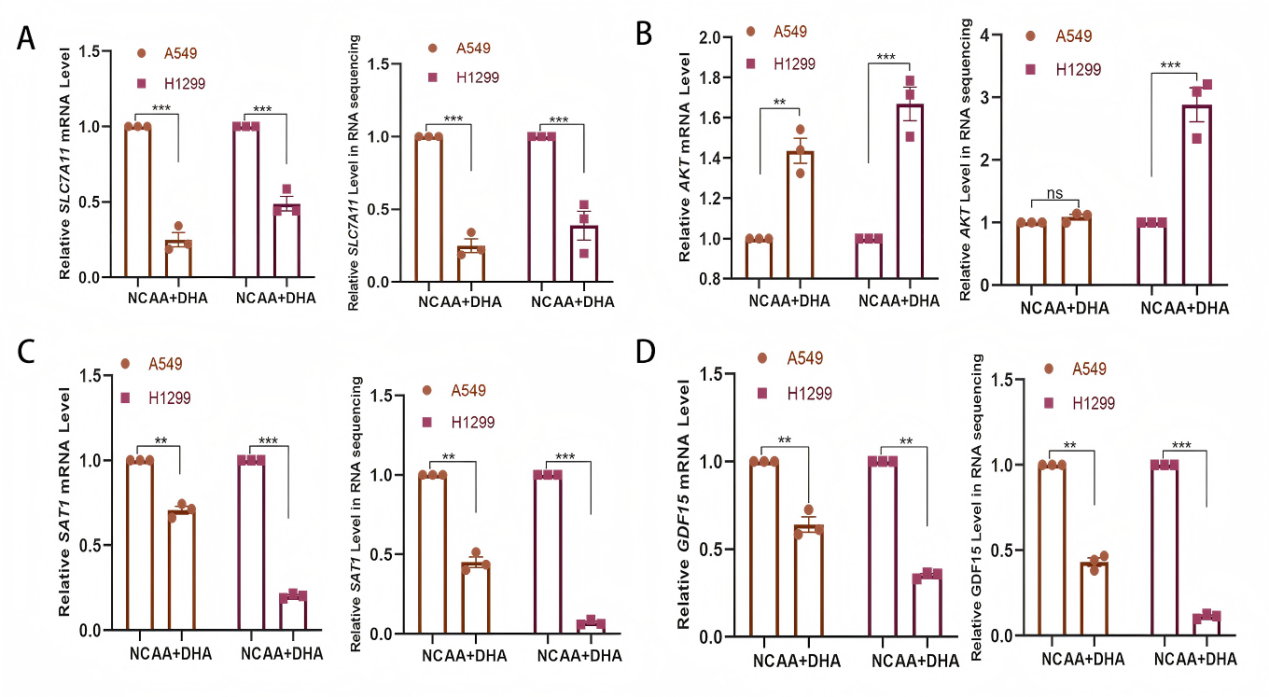


**Supplementary Figure 3.** PCR results were used to validate the RNA sequencing results. (A) Expression of *SLC7A11* in PCR and RNA sequencing. (B) Expression of *AKT* in PCR and RNA sequencing. (C) Expression of *SAT1* in PCR and RNA sequencing. (D) Expression of *GDF15* in PCR and RNA sequencing.

0.001, **** *P* <0.0001.


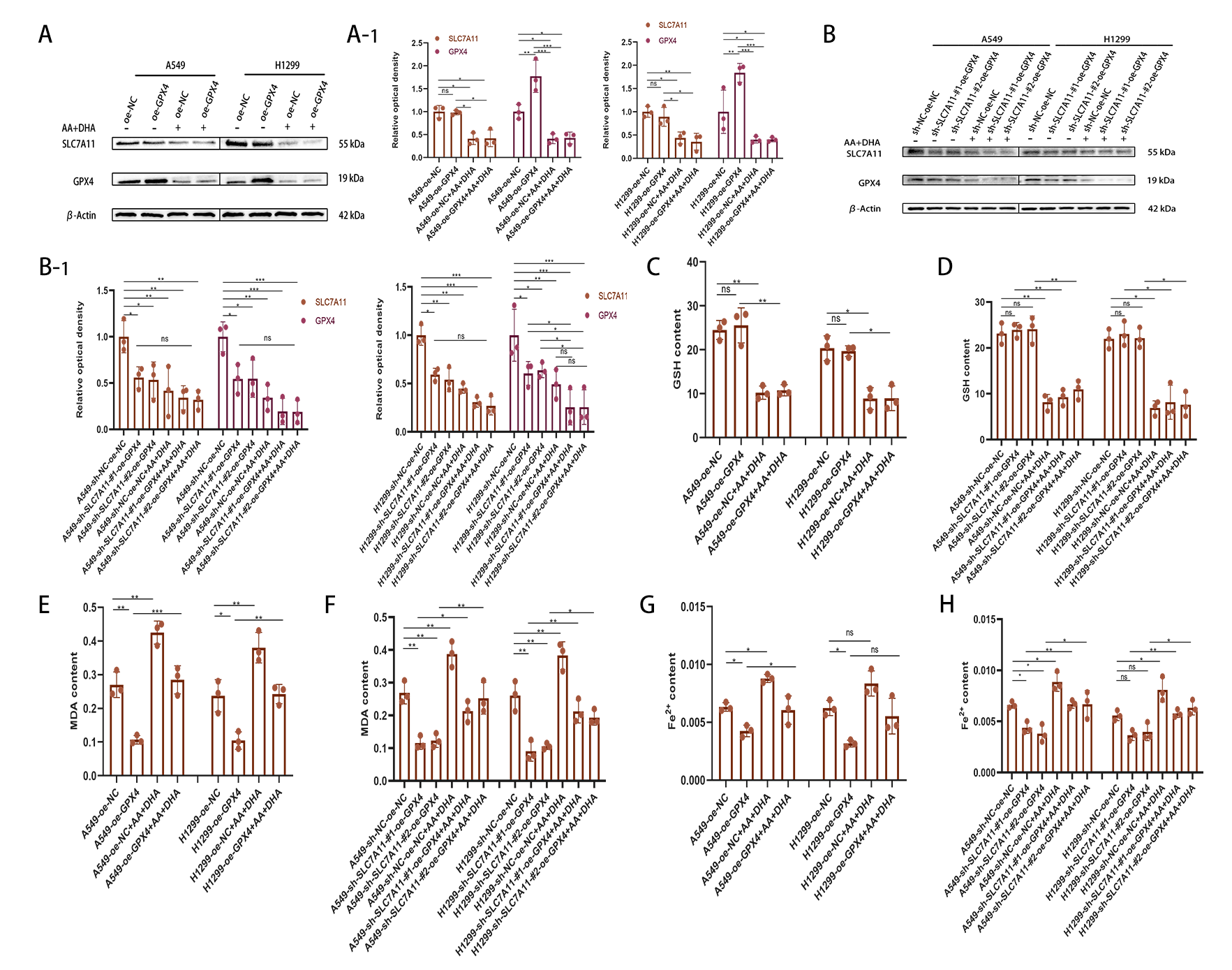


**Supplementary Figure 4.** GPX4 Overexpression Inhibits Ferroptosis and Its Functional Interplay with the SLC7A11-GSH Axis. (A) Protein levels of SLC7A11 and GPX4 were detected by Western blotting in A549 and H1299 cells overexpressing GPX4 under conditions of AA combined with DHA treatment versus untreated conditions. (B) Protein levels of SLC7A11 and GPX4 were detected by Western blotting in A549 and H1299 cells following SLC7A11 knockdown and GPX4 overexpression under conditions of AA combined with DHA treatment versus untreated conditions. (C, E, G) Effects of GPX4 overexpression followed by AA/DHA treatment on key ferroptosis indicators glutathione (GSH), malondialdehyde (MDA), and Fe^2+^ levels. (D, F, H) Effects of GPX4 overexpression combined with SLC7A11 knockdown followed by AA/DHA treatment on ferroptosis indicators GSH, MDA, and Fe^2+^ levels. *P < 0.05, **P < 0.01, ***P < 0.001.
